# Nephrogenic Diabetes Insipidus Affecting Three Males in Two Generations—Case Report and Review of the Literature

**DOI:** 10.3390/children12020195

**Published:** 2025-02-06

**Authors:** Ramona Stroescu, Adela Chiriţă-Emandi, Maria Puiu, Flavia Chisavu, Ruxandra Steflea, Gabriela Doroş, Mihai Gafencu

**Affiliations:** 1 “Louis Turcanu” Children’s Clinical and Emergency Hospital, Iosif Nemoianu 2, 300011 Timisoara, Romania; stroescu.ramona@umft.ro (R.S.); adela.chirita@umft.ro (A.C.-E.); maria.puiu@umft.ro (M.P.); steflea.ruxandra@umft.ro (R.S.); doros.gabriela@umft.ro (G.D.); mgafencu@umft.ro (M.G.); 2 Department XI of Pediatrics, “Victor Babes” University of Medicine and Pharmacy Timisoara, Eftimie Murgu Sq. No. 2, 300041 Timisoara, Romania; 3 Department of Microscopic Morphology, Genetics Discipline, Center of Genomic Medicine, “Victor Babeș” University of Medicine and Pharmacy, 300041 Timisoara, Romania; 4 Centre for Molecular Research in Nephrology and Vascular Disease, Faculty of Medicine “Victor Babes”, “Victor Babes” University of Medicine and Pharmacy Timisoara, 300041 Timisoara, Romania

**Keywords:** nephrogenic diabetes insipidus, infant, treatment

## Abstract

**Background:** Nephrogenic diabetes insipidus (NDI) is defined as the inability of the kidney to concentrate urine owing to the insensitivity of the distal nephron to the antidiuretic hormone, arginine vasopressin. NDI is a heterogeneous rare autosomal dominant or X-linked disease. **Objective:** We present a family with nephrogenic diabetes affecting three males in two generations. **Methods:** We report two boys with NDI: a 4-month-old infant who was treated for fever, vomiting, and failure to thrive, and his 10-year-old uncle (the mother’s brother), who was admitted concurrently for consuming 11 L of fluid per day. According to family history, the mother’s sibling passed away at the age of two from severe hypernatremic dehydration. **Results:** The infant’s clinical and laboratory evaluation revealed a 7.8 mL/kg/h urine output, hypernatremic hyperchloremic alkalosis, extremely low urine density (1002), and elevated copeptin level. In contrast, the uncle’s clinical and laboratory evaluation revealed marked polyuria, low urine density, and elevated copeptin, all of which were suggestive of diabetes insipidus. After starting hydrochlorothiazide treatment (2 mg/kg/body), the infant’s urine production reduced (2.85 mL/kg/h); however, severe hypokalemia and alkalosis followed. Spironolactone, an aldosterone antagonist, were added, with good therapeutic response. Hydrochlorothiazide was administered to the uncle, and his daily fluid intake decreased to 3–4 L. Given the family history, Sanger sequencing for the *AVPR2* variant was performed on the boys and the infant’s mother. Analysis showed hemizygous likely pathogenic variant c.335G>A p. (Cys112Tyr) in the 2 boys and heterozygous (carrier) status of the mother. Within the same family, we observed phenotypic heterogeneity: one child died at the age of two, another lived well into ten years without therapy, and a four month-old baby could have had a poor outcome without specific treatment. **Conclusions:** NDI is a rare and possibly fatal genetic disorder with heterogeneous manifestations. In families with a history of NDI, molecular genetic testing is crucial for family planning.

## 1. Introduction

Nephrogenic diabetes insipidus (NDI) is defined as the inability of the kidney to concentrate urine owing to the insensitivity of the distal nephron to the antidiuretic hormone, arginine vasopressin (AVP). NDI is a heterogeneous rare autosomal dominant or X-linked disease. The late distal tubules and collecting ducts’ inability to react to AVP causes polyuria and polydipsia, which in turn causes electrolyte imbalance and severe dehydration (hypernatremia and hyperchloremia) [1,2]. Summary of laboratory results characteristic of NDI is as follows: urine output (UOP) > 4 mL/kg/h, serum sodium > 170 mmol/L, serum osmolality > 300 mOsm/kg, urine osmolality < 300 mOsm/kg, and a urine specific gravity < 1005. Growth retardation, vomiting or poor feeding, retching, unexplained fever, lethargy or irritability, polydipsia, polyuria, nocturia, or nocturnal enuresis are the primary symptoms in infants and toddlers at presentation. Severe or potentially fatal dehydration and electrolyte imbalance (hypernatremia and hyperchloremia) are also common [3].

Compared to central diabetes insipidus (CDI), NDI is more common in children [4]. Both acquired and hereditary (congenital) forms of NDI are found in youngsters. A pathogenic variant in the genes for aquaporin-2 (*AQP2*) or vasopressin V2 receptor (*AVPR2*) causes congenital NDI. The aquaporin-2 (*AQP2*) water channel is encoded by the *AQP2* gene, whereas the vasopressin V2 receptor is encoded by the AVPR gene [4]. With a frequency of four occurrences per million male births, X-linked NDI accounts for 90% of congenital cases. Congenital NDI has an autosomal dominant or recessive component in the remaining 10% of cases. Significant research efforts have been undertaken over the past 20 years to comprehend NDI at the genetic, cellular, molecular, and biological levels as well as to suggest novel treatment approaches for NDI [5].

## 2. Material and Methods

The data were extracted from the electronic medical records. Anthropometric measurements were noted. Fluid intake was measured during a four hour period and noted based on the water balance (in = out). The blood tests included the hematological parameters, renal, liver, and protein status. Both urine and blood biochemistry measurements were performed to determine the urine and blood osmolality. Also, the blood pressure and heart rate were monitored during admission. Body weight, fluid balance, and constant blood and urine studies were obtained during the water deprivation test and Desmopressin administration. Changes in body weight, plasma sodium, and plasma and urinary osmolality were evaluated every two hours Desmopressin administration was followed by a repeated urine osmolality measurement and volume 4 h after oral administration. Renal imaging was also performed. The family history was obtained from the legal guardian. We report two boys with nephrogenic diabetes insipidus: a 4-month-old infant who was admitted for fever, vomiting, and failure to thrive, and his 10 year-old uncle (the mother’s brother), who was admitted concurrently for consuming 11 L of fluid per day. According to the family history, the mother’s sibling passed away at the age of two from severe hypernatremic dehydration. The dead brother’s information were obtained from the general practitioner. The study was approved by the hospital’s Medical Ethics Committee (140/06 December 2024) in accordance with the World Medical Association’s Ethics Code and informed consent was waived.

## 3. Results

### 3.1. Polyuria and Polydipsia with Normal Development

**Figure 1:**
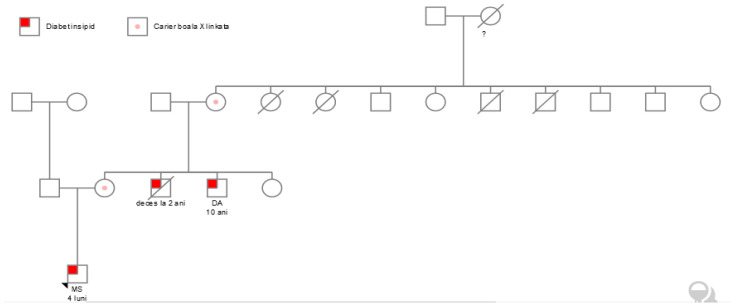
The family tree shows that the 2 male patients, D.A. and M.S, have the disease-causing *AVPR2* variant in hemizygous and the infant’s mother and grandmother are carriers of the variant.

A 10-year-old boy (the uncle), D.A., was admitted to our nephrology department with polyuria and polydipsia. The family history revealed that he was one of the four siblings of the family, and had a brother who died at the age of 2 years old (Figure 1). The history was unremarkable besides recurrent vomiting episodes in the infancy period. The anthropometric data corresponded with the patient’s age, 31 kg (kg) and 133 cm (cm) (15th percentile using the World Health Organization growth curves [6]), BMI = 17.5 kg/square meters (60th percentile) with normal neurological and cognitive development (QI = 110). The physical examination was unremarkable.

Renal ultrasound showed normal kidney size without structural changes. The urine output was measured during hospitalization and revealed marked polyuria (11 L/day = 13.88 mL/kg/day) that was associated with increased water intake (12 L/day). The blood pressure (BP) was slightly elevated for the height and age of the patient (BP = 110/70 mmHg), being below the 90th percentile (optimal 50th percentile BP = 102/61 mmHg) and normal heart rate (HR). The patient had normal hematological, liver, and kidney parameters. There were no electrolytic imbalances at the time of the admission. The patient had normal glycemia (5.64 mmol/L, normal range 3.88–6.38 mmol/L) and insulin levels (8.22 uIU/mL, normal range 2.6–24.9 uIU/mL). In addition, the urine analysis was negative for glucose. The 24 h urine revealed hypotonic and hypoosmolar urine (density = 1001, urine osmolality = 54 mOsm/L), with tubular proteinuria secondary to hyperfiltration (297 mg/24 h).

The next step was represented by the water deprivation test with the following parameters recorded as seen in Table 1. During the test baseline weight, strict fluid balance, urine, and plasma were obtained from the patient while the fluid intake was withheld. The test was performed under strict medical intervention.

At the end of the water deprivation test, the serum parameters were as follows: sodium-156.7 mmol/L, potassium = 4.37 mmol/L, osmolality = 363.25 mOsm/kg (normal range: 275–295 mOsm/kg). The urine output during the 4 h test was approximately 1300 mL with a constant low density of 1005. The blood pressure did not differ during the test (BP ranged from 105 to 110/70 mmHg) and the HR was normal (88–93 beats/minute). Afterwards, the patient received Desmopressin (120 mcg/day) without improvement of the urinary osmolality (84.4 mOsm/L), density (1002), or polyuria, thus excluding a central cause of diabetes insipidus. The low urinary osmolality levels maintained during both the water deprivation test and after Desmopressin administration supported the diagnosis of NDI.

The extremely high copeptin level of 67 ng/L, (normal range < 8 ng/L), along with the recurrent vomiting episodes in the infancy period, the current polyuria with hypotonic urine and polydipsia, and the family history (younger brother who died due to hypernatremic dehydration and the infant nephew) were supporting the suspicion of NDI. Genetic sequencing confirmed the diagnosis of X-linked NDI (Figure 1).

### 3.2. Failure to Thrive in an Infant with Polyuria

A 4-month-old male infant, M.S, was admitted at the same time as his uncle D.A. for failure to thrive, recurrent vomiting, and fever. The infant had no siblings and was born at 40 weeks of gestation through vaginal delivery without significant prenatal or postnatal complications, with a weight of 3100 g and a height of 50 cm. He had been breastfed and provided supplementary feeding on demand. He had also received the appropriate vaccinations on time.

**Figure 2:**
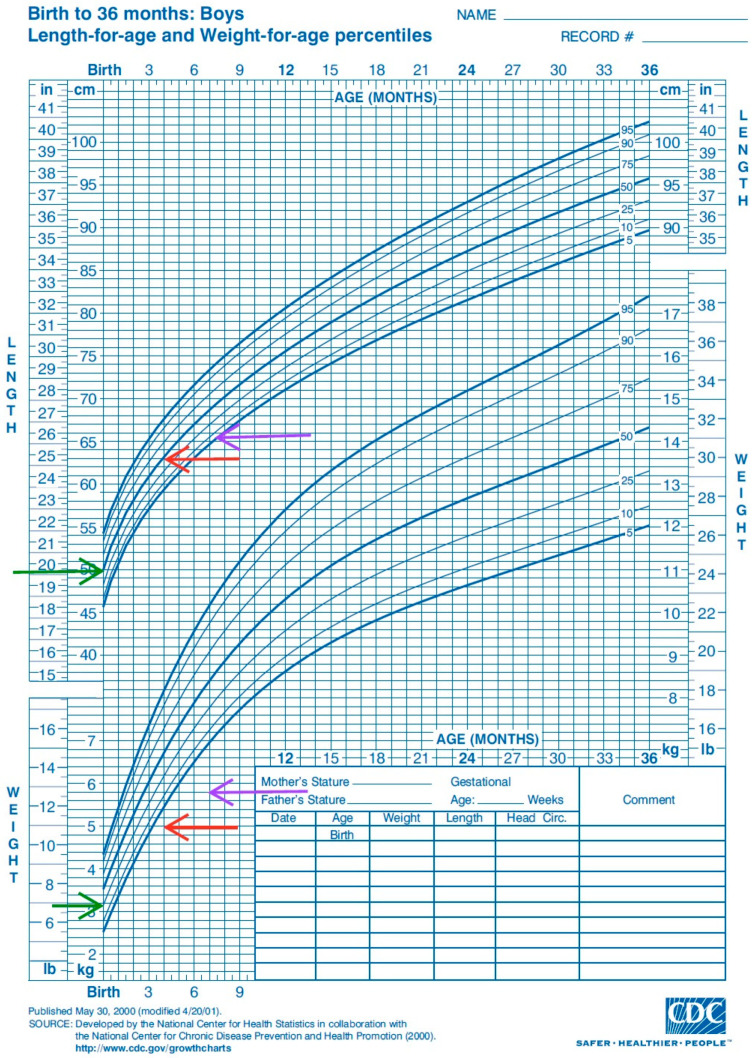
Growth chart of the infant from birth until the last follow-up. Legend: The green arrow points to the weight and height at birth of the infant. The red and purple arrows point to the statural and weight deficit of the infant, with a slight improvement in weight after hospital admission (purple arrow).

The history was marked by a urinary tract infection with *Escherichia Coli*. In the last 2 months, the patient presented postprandial vomiting and growth impairment with failure to thrive even after adjusting the mother’s diet and multiple milk powder formulas. At the time of the admission, the patient had a good general state, ringed, subcutaneous cellular tissue poorly represented, lazy skinfold, and slightly sunken anterior fontanel, with a blood pressure of 83/46 mmHg. The boy’s body weight and height were 5 kg (<3rd percentile) and 65 cm (50th percentile)—Figure 2. The patient had a global developmental delay, as he could not turn from the dorsal to the ventral position. There were no obvious abnormalities in the heart and lungs.

The hematological parameters showed normocytic anemia and leukocytosis with monocytosis in the presence of non-specific inflammatory markers. The renal and liver function tests were within normal range. The electrolytic disturbances showed hyperchloremic hypernatremic metabolic alkalosis, with high serum sodium and chloride levels, 150 mmol/L and 114 mmol/L, respectively (normal range for sodium 135–145 mmol/L and chloride 95–105 mmol/L), with high plasmatic osmolality (335 mOsm/kg H_2_O). The low urinary density of 1010 was followed by a 24 h urine measurement, which revealed marked polyuria (7.1 mL/kg/hour) and low urinary osmolality (85 mOsm/kg H_2_O). The hypernatremic dehydration was due to reduced fluid ingestion with marked urinary losses (milk ingestion was 550 mL/day and urine output was 880 mL/day). The abdominal ultrasound revealed normal kidney size without structural changes and no evidence of pyloric stenosis as an underlying cause of recurrent vomiting. Transfontanel echography was also normal. Because of the patient’s young age, intolerance to excessive thirst, and underlying dehydration status, the water deprivation test could not be conducted. We continued with measuring the copeptin level which was extremely high (56 ng/L) due to renal tubular resistance to vasopressin.

### 3.3. Genetic Testing

Because of the family history, X-linked transmission was considered (Figure 1). The *AVPR2* gene was analyzed by PCR and Sanger sequencing of both DNA strands of the entire coding region and the highly conserved exon–intron splice junctions were performed for the 10-year-old boy, in Centogene laboratory (Rostock, Germany). Analysis showed a hemizygous likely pathogenic variant NM_000054.7:c.335G>A p. (Cys112Tyr). Subsequently, exon 2 of the *AVPR2* gene was analyzed by PCR and sequencing of both DNA strands of the relevant coding region in the infant boy and his mother. The analysis showed the same variant in the hemizygous state in the infant male and the heterozygous state in his mother. This missense variant was classified with likely pathogenic significance. The variant is absent in gnomAD (https://gnomad.broadinstitute.org/), a database that excludes severe pediatric diseases [7]. In silico predictors estimate a damaging effect for the protein (MetaRNN = 0.991, greater than 0.939) [8]. The family history and symptoms are very suggestive of the disease. The variant was previously reported by a laboratory in the Clinvar database (https://www.ncbi.nlm.nih.gov/clinvar/ accessed on 11 November 2024) as likely pathogenic [9].

We note the phenotypic heterogeneity within the same family, one boy deceased at the age of 2 years (without genetic testing), one male who survived well into age 10 years without treatment, and another 4-month male infant that could have died without specific treatment—see Figure 1 with the family tree.

### 3.4. Treatment and Evolution

Treatment with hydrochlorothiazide was initiated (2 mg/kg/body) in both boys, with a reduction in urine output (2.85 mL/kg/h), but severe consecutive alkalosis and hypokalemia in the infant. An aldosterone antagonist (spironolactone 1 mg/kg/day) was added, with good clinical response. The biological parameters improved, from the electrolytic correction of sodium and chloride that over time led to the normalization of the blood osmolality. The goal was obtained when the patient’s diuresis was corrected coupled with an increased fluid intake that led to weight gain (Table 2, Figure 2). The diuretic dose was increased to a maximum of 2.7 mg/kg/day. The infant’s treatment required hypotonic solutions for the correction of dehydration and high doses of amino acids intravenously.

The low-salt diet was included in both boys, with a salt intake of 0.7–1 mg/kg/day. The uncle’s fluid intake was reduced to 3–4 L/day, while urine output decreased to 3 L/day. He required a dose of 75 mg/day of hydrochlorothiazide to obtain the reduction in diuresis.

Unfortunately, the patients were lost to follow-up, except for the 3-month follow-up of the infant. The patient’s legal guardians could not comprehend the severity of the diagnosis even though they previously had a deceased child in the family. Genetic counseling was performed accordingly. Efforts have been made by the medical team in terms of obtaining a follow-up in both boys from the general practitioner, which ultimately failed as the legal guardians refused the regular check-ups.

The older boy, D.A, had normal development as opposed to his nephew who at the 3-month follow-up seemed to recover in weight but remained underdeveloped for his age (Figure 2).

## 4. Discussion

The phenotypic heterogeneity within the same family represents the highlight of this study. The X-linked transmission of a hemizygous likely pathogenic variant in the males had manifested with phenotypic heterogeneity: one child died at the age of two, another lived well into ten years without therapy, and a four-month-old baby could have had a poor outcome without specific treatment.

Recurrent low-grade fever, irritability, nausea, vomiting, feeding difficulties, sluggish weight gain, constipation, and other non-specific clinical symptoms are common in NDI patients. As a result, NDI is frequently misdiagnosed [10].

NDI is a rare renal tubular hereditary disease caused by *AVPR2* or *AQP2* genetic variants. Disease-causing variants in *AQP2* on chromosome 12q13 and *AVPR2* on chromosome Xq28 cause the pathogenesis [11]. Males account for 90% of NDI cases and are characterized by an X-linked recessive inheritance pattern that is primarily familial and results in renal epithelial cells lacking *AVPR2*. To date, more than 60 NDI-causing *AQP2* variants and more than 250 variants in *AVPR2* have been reported [12]. The degree to which polydipsia and polyuria manifest clinically varies widely [13].

Diabetes insipidus includes a large series of disorders. Many of the known disorders are now susceptible to symptomatic treatments or specific interventions such as dietary modification, thiazides, and inhibitors of prostaglandin synthesis. Hydrochlorothiazide (2–4 mg/kg/day) and amiloride (0.3 mg/kg/day) are currently used in treatment; this combination lowers urine volume by 50%, is well tolerated, and can lessen the incidence of hypokalemia [14,15]. Since amiloride frequently results in chronic nausea, indomethacin can be used as a substitute. Patients with NDI may receive varied treatment recommendations and dietary regimens from various physicians. According to a survey, the majority of physicians (93%) recommend thiazide to treat NDI, whereas 62, 55, and 43% recommend amiloride, non-steroidal anti-inflammatory medications, and indomethacin, respectively [16]. These recommendations are currently supported by the international expert consensus statement on the diagnosis and management of congenital NDI [17].

However, these approaches can only ameliorate the clinical phenotype. In infants, treatment is particularly difficult. It aims to avoid episodes of dehydration and obtain a normal growth curve. The thiazide diuretic therapy is based on its paradoxical action: it decreases the reabsorption of Na in the distal convoluted tubule with increasing excretion of Na, causing contraction of extracellular fluid volume, leading to a decreasing glomerular filtration rate (GFR) with increased proximal tubular reabsorption of Na and water. This way, less Na and water reach the distal tubule, and less water is eliminated, resulting in a decrease in urinary output (improvement of polyuria) [18,19].

The main approaches used to treat NDI are the limitation of salt intake, consumption of enough fluids, reduction in water excretion, and correction of hyperosmotic status caused by hypernatremia and hyperchloremia. The suggested treatment typically consists of the following elements: direct activation of cAMP signaling pathways in primary renal cells, AQP2 activation through circumvention of *AVPR2* signal transduction pathways, restoration of the impaired receptor function, and direct improvement of AQP2 function [20]. Numerous recent studies have investigated novel treatment strategies. The correct folding of mutant proteins is facilitated by the restoration of cAMP signaling pathways broken by *AVPR2* variants employing chemical chaperones, glycerol, and dimethyl sulfoxide (unknown mechanism), which allows the misfolded proteins to escape from the endoplasmic reticulum and be produced correctly [21,22]. Chemical and pharmacological chaperons are possible therapeutic approaches in rescuing the plasma membrane expression of functional misfolded mutant proteins [23].

With the help of pharmacological chaperones, the mutant receptors can be moved out of the endoplasmic reticulum to the cell surface. There, they might be capable of binding to their ligands and responding to hormone stimulation by initiating signal transduction [24]. There are studies regarding the use of Tolvaptan, an approved medicine in the treatment of syndrome of inappropriate antidiuretic hormone (SIADH), and rapidly progressing autosomal dominant polycystic kidney disease (ADPKD) as a pharmacological chaperon able to inhibit the basal (constitutive) activity of V2R and it can increase the cell surface expression of the transiently expressed wild-type V2R [25].

Over the past few years, several studies have demonstrated the potential to increase urine concentration in NDI patients, at least in theory, by avoiding faulty *AVPR2* signaling and reestablishing appropriate AQP2 expression at the apical plasma membrane. There are two categories of methods for evading the *AVPR2* signaling pathway that are currently being studied: Cytosolic cAMP activation by suppression of phosphodiesterases (PDE) and activation of additional G pro-tein-coupled receptors (GPCRs) connected to Gs/adenylyl cyclase expressed in the collecting duct (CD) main cells and cAMP-independent pathway activation. The result is restoring proper salt and water homeostasis by activating other GPCRs expressed in the same renal cells expressing defective *AVPR2* [26,27]. A variety of cAMP-independent methods to avoid the *AVPR2*-elicited pathway have been reported in the literature, including cyclic guanosine monophosphate (cGMP) pathway activation (NO, Sildenafil), inhibition of the epidermal growth factor receptor (EGFR) (Erlotinib), AMP-activated protein kinase (AMPK) activation (Metformin), statins, and peroxisomal proliferator-activated receptor subtype γ (PPAR-γ) agonists (Rosiglitazone) [28,29].

This study presents a case of X-linked recessive hereditary NDI affecting three males in two generations that were caused by a rare variant of *AVPR2* with phenotypic heterogeneity within the same family. Therefore, early diagnosis and treatment of NDI are important. Early interventions can prevent dehydration, growth retardation, and other complications. Gene therapy has become a research hotspot, and advances in genetic research will lead to potential novel treatments for NDI. Unfortunately, the parents did not accept the diagnosis and there was no long follow-up of the patients. Several discussions were attempted by the nephrology and genetics teams to ensure the family understood the health risk of not administering the treatment, as well as the recurrence risk for other pregnancies of the carrier females.

## 5. Conclusions

Increased diuresis with persistent hypernatremia and failure to thrive in infancy should raise the suspicion of diabetes insipidus. Anamnesis is important, as well as fluid intake and output. The symptomatic treatment aims to ensure the intake of adequate calories for growth and to avoid severe dehydration. In families with a history of nephrogenic diabetes insipidus, molecular genetic testing is crucial for family planning.

## Figures and Tables

**Table 1:** Water deprivation test followed by Desmopressin administration.

Time	Weight (kg)	Weight Deficit (%)	Diuresis(mL)	Na (mmol/L)	Urine Osmolality (mOsm/kg)	Plasma Osmolality (mOsm/kg)
8 AM	29	-	-	153.4	54	359
10 AM	28	2.3	850	154	60	360
12 AM	27.5	3.27	1300	156.7	84	363.25
4 h post 120 mcg of Desmopressin	27.5	-	-	160	90	365

Legend: kg = kilograms; % = percentages; mcg = micrograms; mL = milliliters; mmol/L = millimoles per liter; mOsm = milliosmoles; Na = sodium.

**Table 2:** Results of laboratory examinations and clinical manifestations of the 4-month-old infant before and after treatment.

Clinical and Laboratory Examinations	At Admission	After 3 Months of Treatment	Normal Values
Urine density	1001	1008	1.008–1.025
Blood osmolality (mOsm/kg)	335	285	280–310
Urine osmolality (mOsm/kg)	85	300	550–1100
Blood sodium (mmol/L)	150.8	138	135–147
Blood chloride (mmol/L).	114	110	95–110
Daily volume of liquid intake (mL)	550	750	-
Daily volume of urine output (mL/kg/h)	7.8	2.85	-
Body height (cm).	65	66	65
Body weight (kg)	5	5.8	7.1

Legend: mOsm/kg = milliosmoles per kilogram; mmol/L = millimoles per liter; cm = centimeters; kg = kilograms; mL = milliliters.

## Data Availability

All the anonymized data can be available at the specific request to the corresponding author at e-mail farkas.flavia@umft.ro. After agreement from all the authors and after the acceptance of the request by the Ethics Committee from Emergency Hospital for Children “Louis Turcanu” from Timisoara, the anonymized data will be shared. We did not upload the data due to security considerations.

## Abbreviations

The following abbreviations are used in this manuscript:

NDI	Nephrogenic diabetes insipidus
CDI	Central diabetes insipidus
AQP2	Aquaporin-2 water channel
AVPR2	Vasopressin V2 receptor
